# Sociodemographic landscape of suspected prostate cancer referrals and diagnoses across North East London

**DOI:** 10.1002/bco2.495

**Published:** 2025-02-04

**Authors:** Muhammad Haider, Jeffrey J. Leow, James S. A. Green, Chamkhor Dhillon, Angela S. Wong, Yin Zhou, Sara Paparini, Benjamin W. Lamb, Prabhakar Rajan, for the North East London Cancer Alliance Urology Expert Reference Group

**Affiliations:** ^1^ Centre for Cancer Cell and Molecular Biology, Barts Cancer Institute Queen Mary University of London London UK; ^2^ Department of Urology Barts Health NHS Trust London UK; ^3^ Department of Urology University College London Hospitals NHS Foundation Trust London UK; ^4^ North East London Cancer Alliance NHS North East London London UK; ^5^ Department of Gastroenterology Barts Health NHS Trust London UK; ^6^ Centre for Cancer Screening, Prevention, and Early Diagnosis, Wolfson Institute of Population Health Queen Mary University of London London UK; ^7^ SHARE Collaborative, Wolfson Institute of Population Health Queen Mary University of London London UK

**Keywords:** ethnicity, healthcare inequality, prostate cancer, social deprivation, 2‐week wait

## Abstract

**Objectives:**

The objective of this study is to identify healthcare inequities in referrals and diagnoses of suspected prostate cancer (PCa) in an ethnically diverse and socially deprived large urban region.

**Methods:**

Retrospective cohort study of 2‐Week Wait (2WW) suspected PCa patients (*n* = 12 947) referred to two acute NHS Trusts in North East London (NEL) from February 2019 to August 2023. Sociodemographics, diagnosis and pretreatment staging data were collected from patient records. We examined referral and diagnosis statistics, age at referral, radiological T‐stage at diagnosis, levels of deprivation by ethnicity and the impact of COVID‐19 pandemic lockdowns on proportion of referrals and diagnoses by ethnicity and T‐stage at diagnosis. Uni‐ and multivariable logistic regression was performed to identify predictors of locally advanced (T‐stage ≥T3) disease.

**Results:**

Of all referrals, 22% were diagnosed with PCa. There were no statistically significant differences in referrals, diagnoses or T‐stage of PCa by ethnicity during COVID lockdown versus non‐lockdown periods (*p* > 0.05). Compared to men from any other ethnicity, Black men (from Black British, Black African and Black Caribbean ethnic groups) were diagnosed at a younger age (mean = 65 years), had the highest age‐adjusted PCa incidence rate of 149 per 100 000 person‐years, were from the most deprived backgrounds, and were diagnosed with the highest proportion of localised PCa (74%). Multivariable analysis of a patient subgroup revealed age bands 71–80 years (OR 2.01, 95% CI 1.31–3.07) and >80 (OR 4.27, 95% CI 2.25–8.08) as independent positive predictors of locally advanced PCa, and Black ethnicity as an independent predictor of localised disease (OR 0.66, 95% CI 0.43–1.00). Limitations of this study include the exclusion of PCa cases diagnosed outside the 2WW pathway, as well as missing data on Prostate‐Specific Antigen (PSA) levels, distant radiological staging and histopathological findings.

**Conclusion:**

We identify disparities in PCa incidence, stage and age at presentation, as well as socio‐economic deprivation among Black men in NEL. Targeted efforts are needed to mitigate these healthcare inequities.

## 1 INTRODUCTION

Prostate cancer (PCa) is the most commonly diagnosed cancer in men in the United Kingdom, with a 1:8 lifetime risk.^1^ Risk is unequally distributed: Black men have twice the incidence (1:4 lifetime risk) and twice the mortality rate compared to White men,^1^ and they tend to develop PCa at a younger age.^2^ Conversely, Asian men have a much lower lifetime risk (1:13) compared with other ethnic groups and lower mortality rates.^1^ The picture is similar in the United States, with higher age‐adjusted incidence and mortality rates for Black men compared with White.^3^

In the United Kingdom, PCa incidence has also been linked to levels of social deprivation, with increasing deprivation being associated with a lower disease incidence.^1^, ^3^ This may be explained by lower socio‐economic status being associated with lower levels of Prostate‐Specific Antigen (PSA) testing,^2^ reduced access to health services in deprived areas^4^ and more patient‐driven PSA testing in less deprived patients.^5^ Lower incidence due to less testing likely explains why, in England, men in the most deprived quintile are more likely to present with metastatic disease (19%) compared to the least deprived quintile (15%).^6^

Based on the 2021 Census, London is the most ethnically diverse region in England and Wales with 46% of residents identifying as from Asian, Black, Mixed or ‘Other’ ethnic minority groups.^7^ The North East London Cancer Alliance (NELCA) brings together regional health and social care organisations to improve cancer care and covers four local authority regions within the top 10 most diverse boroughs in the United Kingdom.^7^ There are high levels of social deprivation and unemployment within North East London (NEL), with two of the boroughs ranking within the top 10 most deprived in the United Kingdom.^8^

Here, we explore sociodemographic patterns within secondary care referrals for suspected PCa across an ethnically diverse population in NEL, to inform targeted interventions to improve early diagnosis of PCa and reduce healthcare inequalities.

## 2 PATIENTS AND METHODS

Data were retrospectively collected on all suspected 2‐Week Wait (2WW) PCa pathway patients referred from primary care to Barts Health NHS Trust (BH) and Barking, Havering and Redbridge University Hospitals (BHRUT) NHS Trusts, who had their first clinic appointments between February 2019 and August 2023. Patients were referred based on an elevated age‐adjusted PSA level using thresholds defined in National Institute for Health and Care Excellence (NICE) guidelines and/or abnormal digital rectal examination.^9^

Data were collected from secondary care electronic records on age, date of first 2WW appointment, Local Authority District (LAD) by patient address, date of PCa diagnosis and pretreatment radiological tumour staging according to the T‐staging^10^ (localised PCa was classified as ≤T2 and locally advanced as ≥T3, distant radiology staging was not available). Self‐reported patient ethnicity data were extracted from primary care records and categorised broadly in accordance with UK Census 2021 criteria (Table S1) to enable comparison with national data. Different ethnic groups in the Census (e.g., Black African, Black Caribbean or Indian and Pakistani) were organised by broader categories (i.e., Black or Asian) for ease of comparison and description. Tumour stage was based on pre‐diagnostic magnetic resonance imaging (MRI) reports or uro‐oncology multidisciplinary team meeting (MDT) outcomes. Data accuracy was confirmed by manual cross‐referencing recorded data against 30 randomly selected individual patient records. Levels of local deprivation at LAD level (matched to the location of the home address) were determined by using the average score from the 2019 Index of Multiple Deprivation (IMD), which is a widely used deprivation score^11^, ^12^ and measures multiple indicators for deprivation across seven domains (income, employment, education, health, crime, barriers to housing and services and living environment).^8^ All patient data were stored on secure hospital servers, and the study protocol received institutional audit approval at BH (ID: 13933).

The referral‐to‐cancer diagnosis conversion rate for each ethnic group was calculated as follows: 100 multiplied by number of patients with a confirmed PCa diagnosis divided by the number of patients referred on 2WW pathway. Age‐adjusted incidence rates for each ethnic group was calculated using the direct method with the 2021 UK England and Wales population as the standard population.^13^ Crude yearly incidence rate for each ethnicity was calculated using patients with a registered home address within the BH catchment boroughs of Newham, Waltham Forest and Tower Hamlets (City of London data was not available). These data were then compared to UK 2021 Census data for the registered male population by age and ethnicity within these three boroughs^14^ and then were multiplied by 3 to estimate the population of each ethnic group across 3 years being examined (2020 to 2022). To enable calculation of yearly incidence rate, instances where the number of PCa cases exceeded the number of registered residents for a particular age band and ethnic group were excluded to enable calculation of an overall age‐adjusted incidence rate for each ethnicity. We then performed a logistic regression analysis to examine predictors of locally advanced (T‐stage ≥T3) disease, adjusting for age, ethnicity and IMD scores for BH patients as age data were not available for BHRUT patients.

Statistical analysis was performed using STAT SE 14.2 (StataCorp, College Station, TX) including Chi‐squared, ANOVA and logistic regression analysis with *p* < 0.05 taken to indicate statistical significance. Graphs and tables were created using Microsoft Excel (version 2403).

## 3 RESULTS

Between February 2019 and August 2023, 12 947 patients (*n* = 4741 and *n* = 8206 in BH and BHRUT, respectively) had their first clinical appointment on the 2WW suspected PCa pathway (Table 1 & Table S2). Of these, 22% patients (16% and 26% at BH and BHRUT, respectively) were diagnosed with PCa.

**TABLE 1:** Suspected prostate cancer (PCa) referrals and confirmed diagnoses by ethnicity at Barts Health NHS Trust (BH) and Barking, Havering and Redbridge University Hospitals NHS Trust (BHRUT).

Ethnicity	White	Black	Asian	Mixed	Any other ethnicity	Unknown	Total	*p*‐Value
Proportion of 2WW referrals	57% (7347)	18% (2314)	16% (2015)	1.6% (213)	3.5% (456)	4.6% (602)	100% (12947)	<0.001
Proportion of population in NEL	47%	14%	30%	5.4%	3.4%	‐	‐	‐
Proportion of confirmed PCa diagnoses	63% (1812)	18.4% (533)	11% (322)	2.1% (61)	2.5% (72)	3.1% (89)	100% (2889)	<0.001
Referral‐to‐diagnosis conversion rate	25%	23%	16%	29%	16%	15%	22%	‐
Proportion of patients with localised disease(≤T2)	61% (840)	74% (318)	61% (157)	65% (34)	71% (41)	71% (47)	64% (1437)	0.006
Proportion of patients with locally advanced disease (≥T3)	39% (542)	26% (110)	39% (100)	35%^18^	29%^17^	29%^19^	36% (806)	0.009
Mean age across all prostate 2WW referrals (SD) (*n* = 5298)	67 (11)	64 (11)	65 (12)	64 (8.9)	64 (10)	64 (11)	‐	<0.001
Mean age for diagnosed PCa (SD) (*n* = 1085)	70 (9.4)	65 (10)	70 (9.7)	68 (6.8)	68 (8.3)	68 (11)	‐	<0.001
Mean (Index of Multiple Deprivation) IMD score by LAD for referrals (SD) (*n* = 4428)	25 (4.6)	27 (3.9)	26 (4.6)	26 (4.5)	26 (4.8)	25 (4.5)	‐	<0.001
Mean IMD score by lad for PCa diagnoses (SD) (*n* = 703)	25 (0.23)	27 (0.29)	26 (0.46)	23 (1.1)	26 (0.91)	26 (1.2)	‐	<0.001

Abbreviations: IMD, Index of Multiple Deprivation; Lad, Local Authority District; NEL, North East London.

**FIGURE 1:**
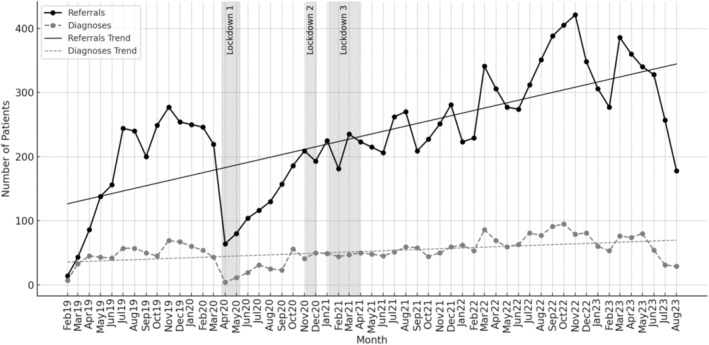
Suspected prostate cancer (PCa) referrals and confirmed diagnoses at Barts Health NHS Trust (BH) and Barking, Havering and Redbridge University Hospitals NHS Trust (BHRUT). The UK lockdown periods shown in this graph were from March 23, 2020, to June 1, 2020 (first lockdown), November 5, 2020, to December 2, 2020 (second lockdown), and January 6, 2021, to March 8, 2021 (third lockdown).

The first coronavirus disease 2019 (COVID‐19) UK national lockdown (23 March to 1 June 2020) coincided with a drop of 72% referrals and 91% of diagnoses across both trusts, for the months of March and April 2020 (Figure 1). The second and third lockdowns led to only minimal reductions in numbers of referrals and diagnoses. Consequently, the lowest mean number of referrals and diagnoses per month (173 and 47 patients, respectively) was observed for the year 2020. However, in the following years (starting in 2021), the number of referrals and diagnoses exceeded pre‐pandemic levels and have continued to increase year‐on‐year.

The three COVID‐19 lockdowns disrupted the 2WW service, leading to a reduction in the mean number of monthly referrals and diagnoses by 37% and 40%, respectively, during the lockdown periods. To assess the impact of these service disruptions by ethnicity, we compared the proportions of referrals and diagnoses across different ethnic groups during lockdown and non‐lockdown periods (Table S3). There were no statistically significant differences in the proportions of referrals, diagnoses or in the proportion of patients diagnosed with localised or locally advanced PCa by ethnicity during lockdown periods compared to non‐lockdown periods (*p* > 0.05).

To better understand ethnic differences in diagnoses, we calculated age‐adjusted PCa incidence rates for ethnicity (Table S4) for BH patients (*n* = 4741), who comprised 36.6% of the overall patients. Black men had the highest age‐adjusted incidence rate (149 per 100 000 person‐years) across all age groups, approximately twice that of White men (83 per 100 000 person‐years) and approximately three times higher than that of Asian men (48 per 100 000 person‐years).

There was a statistically significant difference in referral and diagnosis rates across ethnicities (*p* < 0.001). The majority of referrals were in White and Black men (57% and 18%, respectively) and were referred at rates higher than their respective proportions in the NEL population (Table 1). In contrast, Asian men, who represent 30% of the population, constituted only 16% of referrals. Similarly, for PCa diagnoses, White and Black men were the largest groups, at 63% and 18% respectively; both were greater than their respective proportions in the NEL population. Conversely, Asian men formed a smaller proportion of diagnoses (11%) than their proportion of the population in NEL. Mixed patients (details in Table 1) had the highest referral‐to‐diagnosis conversion rates at 29%, followed by White patients at 25% and Black patients at 23%.

To examine whether differences in age‐adjusted incidence rates could be explained by differences in levels of deprivation for referrals and diagnoses in the BH subgroup, we calculated mean IMD scores by LAD across ethnicities. There were statistically significant differences in the mean IMD scores by ethnicities (*p* < 0.001) across all referrals and among PCa diagnoses. Black men were from the most deprived backgrounds, with mean IMD scores of 27 (3.9) and 27 (0.29) for referrals and diagnoses, respectively. White men were from the least and second‐least deprived backgrounds for referrals and diagnoses, respectively, with mean IMD scores of 25 (4.6) and 25 (0.23).

Out of the 2889 patients diagnosed with PCa, 78% (*n* = 2243) had radiological staging information recorded. The majority of patients (64%) were diagnosed with localised PCa (Table 1); there were statistically significant differences in the rates of diagnosis of localised and locally advanced PCa across ethnicities (*p* < 0.05). Black men diagnosed with highest proportion of localised PCa (74%) of any ethnicity, whereas White men were diagnosed with the highest proportion of locally advanced PCa (39%).

Univariable logistic regression analysis was performed to determine predictors of locally advanced PCa for BH patients using the following variables: ethnicity, average IMD score and age (Table 2). The analysis revealed that increasing age was a predictor of locally advanced PCa (OR 1.95, 95% CI 1.32–2.87, 71–80 vs. 61–70) and (OR 4.39, 95% CI 2.39– 8.15 > 80 vs. 61–70), and Black ethnicity was an independent predictor of localised disease (OR 0.56, 95% CI 0.38–0.82 Black vs. White men). Multivariable analysis revealed that increasing age (OR 2.01, 95% CI 1.31–3.07, 71–80 vs. 61–70) and (OR 4.27, 95% CI 2.25–8.08, >80 vs. 61–70) remained independent positive predictors of locally advanced PCa, and Black ethnicity remained an independent predictor of localised disease (OR 0.66, 95% CI 0.43–1.00 Black vs. White men).

**TABLE 2:** Uni‐ and multivariate cc‐advanced prostate cancer (PCa) for Barts Health NHS Trust (BH) patients.

	Univariable analysis		Multivariable analysis	
	Odds ratio	*p*‐Value	Odds ratio	*p*‐Value
Ethnicity				
White	1	N/A	1	N/A
Black	0.56 (0.38–0.82)	0.003	0.66 (0.43–1.00)	0.048
Asian	1.46 (0.90–2.37)	0.13	1.55 (0.91–2.63)	0.11
All other Ethnicities	0.66 (0.34–1.27)	0.2	0.67 (0.32–1.43)	0.3
Average Index of Multiple Deprivation (IMD) Score	0.99 (0.95–1.03)	0.59	1.02 (0.98–1.06)	0.3
Age				
<50	0.66 (0.25 to 1.76)	0.4	0.70 (0.26 to 1.89)	0.5
51–60	0.90 (0.56 to 1.44)	0.7	0.94 (0.57 to 1.54)	0.8
61–70	1	N/A	1	N/A
71–80	1.95 (1.32 to 2.87)	<0.001	2.01 (1.31 to 3.07)	<0.001
>80	4.42 (2.39 to 8.15)	<0.001	4.27 (2.25 to 8.08)	<0.001

## 4 DISCUSSION

We explored ethnicity and social deprivation patterns within suspected PCa 2WW referrals in NEL during a period that coincided with the COVID‐19 pandemic. National data indicate that the largest drop in the number of PSA tests, referrals and diagnoses for suspected PCa occurred during the first lockdown, with less disruption observed during the second and third lockdowns.^15^ Similarly, we found that during the first lockdown, there was a significant reduction in the number of referrals and diagnoses, likely due to difficulty in accessing primary care referral pathways and prioritisation of confirmed cancers in secondary care. The second and third lockdown's impacts were less severe with minimal reductions in the number of referrals and diagnoses. There were no statistically significant differences in referral rates, diagnoses or PCa stages at diagnosis by ethnicity during the lockdown periods. This suggests that whilst COVID‐19 mitigation measures were not successful in maintaining pre‐lockdown patient numbers, disruptions to the 2WW service did not appear to disproportionately affect any ethnic group.

NEL is one of the most ethnically diverse regions in the United Kingdom, where the majority (53%) of residents are from an ethnic minority background.^16^ Ethnicity‐related terminology is inherently complex, and we acknowledge the limitations of using broader ethnic classifiers in our analyses.^17^ However, using broad ethnic groups mitigates the risk of bias from poor recording or lack of self‐reporting, for example, in a scenario where a patient does not specify a particular ethnic subgroup. Additionally, second‐generation immigrants may identify with a more with a broader ethnic group, such as ‘Asian’ instead of specific subgroups such as ‘Indian’ and may also identify with a group related to the host community, such as ‘British‐Asian’.^18^

Consistent with previously published data,^19^ we found that Black men were disproportionately affected by PCa with an age‐adjusted incidence rate almost twice that of White men and three times that of Asian men. Black men were also diagnosed at a younger age compared to other ethnic groups; this is in keeping with previous reports from North West London,^20^ which also has a large ethnic minority population.^18^ In the United Kingdom, increasing deprivation has been shown to be associated with a lower incidence of PCa,^1^, ^3^ which may be partly explained by the association between higher IMD scores and lower levels of PSA testing.^1^ Thus, the fact that Black men have the highest age‐adjusted incidence rate, despite also having the highest levels of deprivation, further highlights the increased risk of PCa in this group.

Black men constituted a similar percentage of referrals compared to their overall ethnic representation within NEL (18% vs. 14%, respectively), suggesting equitable access to the 2WW pathway. Notably, among those referred, Black men had a lower referral‐to‐diagnosis conversion rate compared to White men (23% vs. 25%, respectively). At diagnosis, Black men also had the highest percentage of localised disease (74%) among all ethnic groups. In multivariable analysis, Black ethnicities were the only statistically significant negative predictors of locally advanced disease. Our observations may be explained by well‐informed primary care physicians having a lower age threshold for secondary care referral for Black men, leading to an earlier diagnosis at a younger age. A caveat, however, is that our study did not include men diagnosed outside the 2WW pathway. Since Black men are more likely to be diagnosed in emergency settings,^21^ men with advanced metastatic disease at diagnosis may be missed from our analyses. On the other hand, Asian men, however, who represent 30% of the NEL population, constituted only 16% of referrals and only diagnoses (11%) suggesting a lack of equitable access to the 2WW pathway. Taken together, these data highlight potential healthcare inequities in PCa referrals and diagnoses for men in both Black and Asian ethnic groups.

Consistent with national data that show men over 80 years are more likely to be diagnosed with metastatic disease,^6^ we found that on multivariable analysis age over 80 was the strongest predictor of locally advanced PCa on presentation. In contrast to national trends, which indicate that increasing deprivation is linked to a greater proportion of patients presenting with metastatic disease at diagnosis,^6^ we found that increasing IMD score by LAD was not associated with locally advanced PCa. However, this finding must be interpreted with caution as LADs cover large geographical areas with significant intraregional deprivation variation. More granular data, such as deprivation scores covering smaller geographical areas like Lower Layer Super Output Areas (LSOAs), may be required to better understand the discrepancy between our dataset and national trends. Furthermore, the IMD measures deprivation across the overall population within a LAD and does not take age, sex or ethnicity into account. Previous research has shown that there can be significant variation in levels of deprivation across ethnicities within a LAD, with measures such as the Ethnic Group Deprivation Index (EGDI) potentially providing a more accurate reflection of deprivation.^17^

Our study has several limitations due to missing data. First, our dataset excludes information from a major secondary care provider in NEL, which may lead to an underestimation of incidence rates across ethnic groups. Additionally, there was a lack of age data from BHRUT, resulting in the exclusion of BHRUT patients from the regression analysis. Furthermore, we were missing complete histopathological tumour grade and distant radiological staging data, both clinically important factors associated with survival rates, as evidenced by the relative 5‐year survival rates for localised, regional and distant spread PCa being 100%, 100% and 36.6%, respectively.^21^ The absence of PSA data prevented us from assessing whether current referral thresholds are equally applicable across ethnicities. Lastly, the exclusion of PCa diagnoses outside the 2WW pathway likely led to an underestimation of advanced disease.

## 5 CONCLUSIONS

Our study highlights ethnic disparities in 2WW PCa referrals and diagnoses in NEL predominantly affecting Black men with the highest age‐adjusted incidence rate, diagnoses at younger ages and highest mean IMD score by LAD. Further research on the reasons underpinning ethnic differences in incidence, risk and referrals are urgently needed to reduce healthcare inequities in the region.

## AUTHOR CONTRIBUTIONS

The authors listed below have made substantial contributions to the intellectual content of the paper in the sections described below: *Conception and design*: Muhammad Haider, Chamkhor Dhillon, Benjamin W. Lamb and Prabhakar Rajan. *Acquisition of data*: Muhammad Haider and Chamkhor Dhillon. *Analysis and interpretation of data*: Muhammad Haider, Jeffrey J Leow, Yin Zhou and Prabhakar Rajan. *Drafting of manuscript*: Muhammad Haider, Jeffrey J Leow and Prabhakar Rajan. *Critical revision of the manuscript*: James S.A. Green, Angela S. Wong, Yin Zhou and Benjamin W Lamb. *Statistical Analysis*: Muhammad Haider, Jeffrey J Leow and Yin Zhou. *Obtaining funding*: James S.A. Green, Angela S. Wong, Benjamin W. Lamb and Prabhakar Rajan. *Administrative support, technical or material support*: James S.A. Green, Chamkhor Dhillon and Angela S. Wong. *Supervision*: Prabhakar Rajan.

## CONFLICT OF INTEREST STATEMENT

BL receives honoraria for public speaking from Parsek UK Ltd, consultancy fees from Digital Surgery Ltd, MDoutlook; and honoraria from AstraZeneca PLC and Astellas Pharma Europe Ltd, SP has received funding from Gilead Science and ViiV Healthcare for research unrelated to this article.

## Supporting information


**Table S1.** Ethnic group classifications according to the UK Census 2021.

**Table S2.** Suspected prostate cancer (PCa) referrals and confirmed diagnoses by ethnicity for Barts Health NHS Trust (BH) and Barking, Havering and Redbridge University Hospitals NHS Trust (BHRUT).

**Table S3.** Proportions of referrals and diagnoses of PCa during the COVID lockdown periods at Barts Health NHS Trust (BH) and Barking, Havering and Redbridge University Hospitals NHS Trust (BHRUT). Lockdowns were implemented during the following time periods: March 23, 2020, to June 1, 2020 (first lockdown), November 5, 2020, to December 2, 2020 (second lockdown), and January 6, 2021, to March 8, 2021 (third lockdown).

**Table S4.** Age‐adjusted incidence of prostate cancer (PCa) across ethnicities at Barts Health NHS Trust (BH).

**Data S1.** Supporting Information.
