# Supplementary material for: Sociodemographic landscape of suspected prostate cancer referrals and diagnoses across North East London
Source: BJUI Compass. 2025 Feb 4;6(2):e495. doi: 10.1002/bco2.495 (PMC11794234; doi:10.1002/bco2.495)
Supplement: Supplementary file 1 — Table S1. Ethnic group classifications according to the UK Census 2021. [file BCO2-6-e495-s002.docx]

**Supplementary table 1.** Ethnic group classifications according to the UK Census 2021

| **High Level Ethnicity Group** | **Detailed Breakdown** |
| --- | --- |
| Any Other Ethnicity | Other - Any Other Ethnic Group |
| Asian | Asian - Any Other Asian Background, Asian or Asian British - Pakistani, Asian or Asian British - Indian, Asian or Asian British - Bangladeshi, Other - Chinese |
| Black | Black or Black British - Caribbean, Black - Any Other Black Background, Black or Black British - African |
| Mixed | Mixed - Any Other Mixed Background, Mixed - White and Black African, Mixed - White and Black Caribbean, Mixed - White and Asian |
| Unknown | Patient Refused, Other - Not Stated |
| White | White - British, White - Any Other White Background, White - Irish |
