# Supplementary material for: Sociodemographic landscape of suspected prostate cancer referrals and diagnoses across North East London
Source: BJUI Compass. 2025 Feb 4;6(2):e495. doi: 10.1002/bco2.495 (PMC11794234; doi:10.1002/bco2.495)
Supplement: Supplementary file 2 — Table S2. Suspected prostate cancer (PCa) referrals and confirmed diagnoses by ethnicity for Barts Health NHS Trust (BH) and Barking, Havering and Redbridge University Hospitals NHS Trust (BHRUT). [file BCO2-6-e495-s005.docx]

Supplementary Table 2: Suspected prostate cancer (PCa) referrals and confirmed diagnoses by ethnicity for Barts Health NHS Trust (BH) and Barking, Havering and Redbridge University Hospitals NHS Trust (BHRUT).

|  | BH | | BHRUT | | Total number of referrals | Total number of diagnoses | BH | | | BHRUT | | |
| --- | --- | --- | --- | --- | --- | --- | --- | --- | --- | --- | --- | --- |
|  | Number of referrals | Number of PCa diagnoses | Number of referrals | Number of PCa diagnoses of PCa |  |  | Number of patients with localised PCa (≤T2) | Number of patients with locally advanced PCa (≥T3) | Number of patients with unknown staging | Number of patients with localised PCa (≤T2) | Number of patients with locally advanced PCa (≥T3) | Number of patients with unknown staging |
| White | 2214 | 394 | 5133 | 1418 | 7347 | 1812 | 180 | 150 | 64 | 660 | 392 | 366 |
| Black | 1200 | 222 | 1114 | 311 | 2314 | 533 | 124 | 58 | 40 | 194 | 52 | 65 |
| Asian | 777 | 98 | 1238 | 224 | 2015 | 322 | 37 | 45 | 16 | 120 | 55 | 49 |
| Mixed | 79 | 17 | 134 | 44 | 213 | 61 | 6 | 7 | 4 | 28 | 11 | 5 |
| Any Other Ethnicity | 254 | 23 | 202 | 49 | 456 | 72 | 12 | 7 | 4 | 29 | 10 | 10 |
| Unknown | 217 | 18 | 385 | 71 | 602 | 89 | 11 | 2 | 5 | 36 | 17 | 18 |
| Total | 4741 | 772 | 8206 | 2117 | 12947 | 2889 | 370 | 269 | 133 | 1067 | 537 | 513 |
