# Supplementary material for: Sociodemographic landscape of suspected prostate cancer referrals and diagnoses across North East London
Source: BJUI Compass. 2025 Feb 4;6(2):e495. doi: 10.1002/bco2.495 (PMC11794234; doi:10.1002/bco2.495)
Supplement: Supplementary file 3 — Table S3. Proportions of referrals and diagnoses of PCa during the COVID lockdown periods at Barts Health NHS Trust (BH) and Barking, Havering and Redbridge University Hospitals NHS Trust (BHRUT). Lockdowns were implemented during the following time periods: March 23, 2020, to June 1, 2020 (first lockdown), November 5, 2020, to December 2, 2020 (second lockdown), and January 6, 2021, to March 8, 2021 (third lockdown). [file BCO2-6-e495-s001.docx]

| Ethnicity | Proportion of referrals during non-lockdown periods | Proportion of referrals during lockdown periods | Proportion of diagnoses during non-lockdown periods | Proportion of diagnoses during lockdown periods | Proportion of localised disease (≤T2) during non-lockdown periods | Proportion of localised disease (≤T2) during lockdown periods | Proportion of locally-advanced disease (≥T3) during non-lockdown periods | Proportion of locally-advanced disease (≥T3) during lockdown periods |
| --- | --- | --- | --- | --- | --- | --- | --- | --- |
| White | 57% (6870) | 58% (477) | 40% (1708) | 38% (104) | 59% (801) | 57% (39) | 67% (515) | 68% (27) |
| Black | 18% (2177) | 17% (137) | 23% (964) | 22% (61) | 22% (303) | 22% (15) | 14% (108) | 5.0% (2) |
| Asian | 16% (1885) | 16% (130) | 24% (1035) | 28% (77) | 11% (147) | 15% (10) | 12% (93) | 18% (7) |
| Other | 9.9% (1197) | 9.0% (74) | 14% (581) | 12% (34) | 8.6% (117) | 7.2% (5) | 6.5% (50) | 10% (4) |
| P value |  | 0.6 |  | 0.5 |  | 0.8 |  | 0.3 |

Supplementary Table 3. Proportions of referrals and diagnoses of PCa during the COVID lockdown periods at Barts Health NHS Trust (BH) and Barking, Havering and Redbridge University Hospitals NHS Trust (BHRUT). Lockdowns were implemented during the following time periods: March 23, 2020, to June 1, 2020 (first lockdown), November 5, 2020, to December 2, 2020 (second lockdown), and January 6, 2021, to March 8, 2021 (third lockdown).
