# Supplementary material for: Sociodemographic landscape of suspected prostate cancer referrals and diagnoses across North East London
Source: BJUI Compass. 2025 Feb 4;6(2):e495. doi: 10.1002/bco2.495 (PMC11794234; doi:10.1002/bco2.495)
Supplement: Supplementary file 4 — Table S4. Age‐adjusted incidence of prostate cancer (PCa) across ethnicities at Barts Health NHS Trust (BH). [file BCO2-6-e495-s004.docx]

|  | **White** | | | **Black** | | | **Any Other Ethnicity** | | | **Asian** | | |
| --- | --- | --- | --- | --- | --- | --- | --- | --- | --- | --- | --- | --- |
| **Age Band** | **PCa diagnoses** | **Registered UK 2021 Census population*4** | **Age-adjusted incidence rate per 100,000** | **PCa diagnoses** | **Registered UK 2021 Census population*4** | **Age-adjusted incidence rate per 100,000** | **PCa diagnoses** | **Registered UK 2021 Census population*4** | **Age-adjusted incidence rate per 100,000** | **PCa diagnoses** | **Registered UK 2021 Census population*4** | **Age-adjusted incidence rate per 100,000** |
| **0-4** | 0 | 35505 | 0 | 0 | 14430 | 0 | 0 | 20265 | 0 | 0 | 43815 | 0 |
| **5-9** | 0 | 27105 | 0 | 0 | 16725 | 0 | 0 | 19890 | 0 | 0 | 44520 | 0 |
| **10-14** | 0 | 22620 | 0 | 0 | 19080 | 0 | 0 | 20145 | 0 | 0 | 42600 | 0 |
| **15-19** | 0 | 20595 | 0 | 0 | 19140 | 0 | 0 | 19050 | 0 | 0 | 40995 | 0 |
| **20-24** | 0 | 44235 | 0 | 0 | 18540 | 0 | 0 | 29295 | 0 | 0 | 45735 | 0 |
| **25-29** | 0 | 82005 | 0 | 0 | 18300 | 0 | 0 | 47970 | 0 | 0 | 47235 | 0 |
| **30-34** | 0 | 87810 | 0 | 0 | 15450 | 0 | 0 | 51975 | 0 | 0 | 48495 | 0 |
| **35-39** | 0 | 68865 | 0 | 0 | 12180 | 0 | 0 | 43260 | 0 | 0 | 46530 | 0 |
| **40-44** | 0 | 51825 | 0 | 2 | 11205 | 0.57 | 0 | 36180 | 0 | 1 | 43665 | 0 |
| **45-49** | 6 | 39795 | 0.32 | 9 | 11055 | 1.7 | 0 | 27735 | 0 | 4 | 36615 | 0.35 |
| **50-54** | 28 | 35370 | 1.8 | 26 | 13185 | 5.3 | 2 | 21285 | 0 | 6 | 27225 | 0.51 |
| **55-59** | 49 | 27645 | 5.6 | 49 | 12660 | 8.0 | 5 | 13770 | 1.0 | 11 | 18300 | 0 |
| **60-64** | 86 | 22650 | 9.2 | 62 | 8415 | 15 | 6 | 8295 | 0.70 | 16 | 15060 | 1.5 |
| **65-69** | 110 | 16920 | 9.5 | 52 | 4095 | 32 | 12 | 4605 | 3.2 | 28 | 10785 | 6.3 |
| **70-74** | 112 | 14820 | 21 | 35 | 2370 | 35 | 9 | 2760 | 3.5 | 27 | 4635 | 11 |
| **75-79** | 107 | 9270 | 19 | 28 | 1575 | 30 | 5 | 1425 | 4.8 | 14 | 2775 | 11 |
| **80-84** | 48 | 5295 | 8.2 | 24 | 1980 | 9.2 | 2 | 645 | 3.5 | 13 | 3120 | 8.0 |
| **85-89** | 27 | 2955 | 7.3 | 10 | 915 | 5.6 | 2 | 105 |  | 3 | 1275 | 2.0 |
| **90+** | 6 | 1410 | 1.2 | 3 | 195 | 5.9 | 0 | 0 |  | 3 | 255 | 6.8 |
|  | **Overall Age-Adjusted Incidence Rate per 100,000** | | **83** | **Overall Age-Adjusted Incidence Rate per 100,000** | | **149** | **Overall Age-Adjusted Incidence Rate per 100,000** | | **17** | **Overall Age-Adjusted Incidence Rate per 100,000** | | **48** |

Supplementary Table 4. Age-adjusted incidence of prostate cancer (PCa) across ethnicities at Barts Health NHS Trust (BH).
