# Supplementary material for: Sociodemographic landscape of suspected prostate cancer referrals and diagnoses across North East London
Source: BJUI Compass. 2025 Feb 4;6(2):e495. doi: 10.1002/bco2.495 (PMC11794234; doi:10.1002/bco2.495)
Supplement: Supplementary file 5 — Data S1. Supporting Information. [file BCO2-6-e495-s003.docx]

**Supplementary Material 5**

**North East London Urology Expert Reference Group (ERG):**

**Chairman:** Professor James Green (Barts Health NHS Trust Health)

**Urologists:** Professor Prabhakar Rajan and Mr Benjamin Lamb (Barts Health NHS Trust Health), Miss Jhumur Pati (Homerton Healthcare NHS Foundation Trust) Mr Shailesh Kulkarni (Barking, Havering and Redbridge University Hospitals NHS Trust)

**Oncologist:** Dr Karen Tipples (Barts Health NHS Trust)

**Clinical Scientist:** Funmi Akinlade (Barking, Havering and Redbridge University Hospitals NHS Trust)

**Urology Lead Nurse:** Paula Allchorne (Barts Health NHS Trust)

**Urology Clinical Nurse Specialists:** Sally Buttleman (Barts Health NHS Trust Health), Sacha Ali (Homerton University Hospital NHS Foundation Trust)

**Cancer Performance Manager:** Charlotte Rowe (Homerton Healthcare NHS Foundation Trust)

**Primary Care Lead:** Robert Palmer (North East London Cancer Alliance)

**Urology Service Managers:** Sophia Clarke (Barts Health NHS Trust), Patricia Murphy (Barking, Havering and Redbridge University Hospitals NHS Trust), Cait Kielty-Adey (General Urology Service Manger)

**Senior Project Manager:** Navneet Deol (North East London Cancer Alliance), Nikki Poland (North East London Cancer Alliance), Rosie O’Dea (North East London Cancer Alliance)

**Project Officer:** Esther Akinyosoye (North East London Cancer Alliance)

**ED Programme Manager:** Saira Parker-Deekes (North East London Cancer Alliance)

**AGM for Cancer Performance and Improvement:** Helen Monahan (Barts Health NHS Trust)

**Lead Uro-Onc Nurse:** Samantha Strumeier (Barts Health NHS Trust)

**Programme Manager**: Ellen Quinney (North East London Cancer Alliance), Obinwanne Oniyah (North East London Cancer Alliance)

**Programme Lead:** Wayne Douglas (North East London Cancer Alliance), Sarita Yaganti (North East London Cancer Alliance), Caroline Cook (North East London Cancer Alliance)

**General Manager Cancer Performance/Cancer Lead and Interim Senior Management:** Janet Robinson (North East London Cancer Alliance)

**Communication and Engagement Manager:** Paul Thomas (North East London Cancer Alliance)
